# Supplementary material for: Are carbon emissions trading and green financial instruments synergistic? -Comprehensive quantitative research based on content analysis
Source: PLoS One. 2024 Mar 7;19(3):e0298601. doi: 10.1371/journal.pone.0298601 (PMC10919601; doi:10.1371/journal.pone.0298601)
Supplement: S1 Data — (ZIP) [file pone.0298601.s001.zip › Data/地级市Z_F工作报告.zip!/╕ú╜¿/╕ú╓▌╩╨/└┤╫╘╣1⁄2╓┌║┼í╛┬φ┐╦╩2╛▌═°í┐_2019.docx]

　各位代表：

　　现在，我代表福州市人民政府，向大会报告政府工作，请予审议，并请市政协各位委员提出意见。

　　一、2018年工作回顾

　　2018年，首届数字中国建设峰会在福州成功举办，习近平总书记发来贺信，给全市人民以巨大鼓舞。一年来，在省委、省政府和市委的正确领导下，市政府以习近平新时代中国特色社会主义思想为指导，全面贯彻党的十九大和十九届二中、三中全会精神，凝聚力量、迎难而上、奋力拼搏，推动高质量发展落实赶超，各项工作都取得了新的进展。预计全市地区生产总值7750亿元，增长8.5%左右；一般公共预算总收入1118亿元，同口径增长8.7%；地方一般公共预算收入680亿元，增长7.3%；固定资产投资增长12%；出口总额1642亿元，增长11%；实际利用外资56亿元；社会消费品零售总额4682亿元，增长11.6%；城镇居民人均可支配收入44250元，增长8%；农村居民人均可支配收入19472元，增长9%；居民消费价格总水平上涨1.4%；城镇登记失业率2.46%。完成省下达的减排降碳任务。荣获中国软件特色名城、全国黑臭水体治理示范城市、全国森林旅游示范市、“健康中国”年度标志城市等称号，数字经济发展和土地利用工作受到国务院通报表扬。一年来的工作主要体现在：

　　（一）项目带动持续增强。通过“抓项目促发展”专项行动，清理批而未供土地2.6万亩，处置供而未建土地4635亩，动工建设申远新材料二期、康乃尔MDI等项目2230个，建成投产山力化纤、科乐通三期等项目962个，在全省“五个一批”项目综合考评中位居前列。通过“招商2018”专项行动，引进京东方柔性显示、祥鑫汽车轻量化、依图科技区域总部等项目4139个，其中100亿元以上项目6个。通过“项目落地百日冲刺”专项行动，动建宜家家居、博纳锂电子等项目47个，建成中铝铝精深加工一期、金强建材二期等项目15个，完成大东海绿色精品钢铁、罗源三钢产能置换等技改项目12个。通过“三产跃升季”专项行动，落地服务业项目331个，动建高意亚太区域总部、三江口文旅综合体等项目160个，建成富邦总部、百鲜海峡冷链物流中心等项目127个。

　　（二）产业转型步伐加快。出台36条民营企业扶持措施，落实21项121条惠企政策。新兴产业快速发展，新增国家级高新技术企业285家、科技小巨人领军企业99家、省市级众创空间35家，东南大数据产业园新增关联企业91家，马尾物联网产业基地新增关联企业52家，首届数字峰会期间与阿里、腾讯、京东等签订数字经济项目64项、总投资746.9亿元，国家自主创新示范区福州片区推出两批13项改革创新举措，福州软件园扩建6个分园，通过“榕博汇”等活动引进高层次人才1723人，R&D经费投入强度从1.98%提高至2.18%。传统产业不断壮大，规上工业增加值增长8.9%，民间投资增长28%，技改投资增长50%，13家企业进入中国民营企业500强，新增2家百亿企业，10家企业进入上市程序，恒申集团成为全球最大的己内酰胺生产企业。国家服务业综合改革试点稳步推进，服务业增加值增长9%，新增企业总部86家，东街口、永嘉天地、东二环泰禾等商圈持续提升，海西现代金融中心区加快建设，马尾基金小镇和鼓楼数字产业基金小镇管理规模超过1500亿元，网易考拉等一批跨境电商项目成功落地，盒马鲜生、超级物种、朴朴等新零售业态蓬勃发展，市区移动支付覆盖率达95%，承办第二届世遗博览会、第八届中国博博会等各类展会活动90场，改造提升鼓岭国家级旅游度假区，建成永泰欧乐堡海洋世界、闽清瓷天下等旅游项目，全市接待游客8250万人次、增长22.4%。

　　（三）乡村振兴扎实推进。全市农林牧渔业总产值增长3.7%，粮食生产保持稳定。特色现代农业稳步发展，新建设施农业3000亩，新增国家数字农业试点项目1个、省级现代农业产业基地17个。现代渔业加快转型，出台10条鼓励远洋渔业发展措施，新建马来西亚水产养殖基地，获批建设南极磷虾捕捞船2艘，“振鲍1号”新型养殖平台成功下水。农村基础设施不断完善，新建改造农村公路166.9公里，乡镇污水处理设施、行政村生活垃圾无害化处理实现全覆盖，84个重大水利项目完成年度投资计划，晋安区获评全国“四好农村路”示范区。打造美丽乡村300个，提升美丽乡村示范村100个，美丽乡村覆盖率位居全省第1。拆除“两违”1246万平方米。整治高速公路、高铁沿线人居环境686公里，治理水土流失13.6万亩。

　　（四）城市品质日益提升。新城建设全面提速，长乐滨海新城核心区城市设计等47项规划编制完成，数字中国会展中心、新城综合医院等227个项目加快建设，数字福建商务云、水上运动中心等17个项目建成投用；仓山三江口片区市一医院新院、南台岛环岛路等121个项目有序展开，海峡文化艺术中心建成开放；马尾琅岐岛大型文旅项目开工建设。老城提升快速推进，建成“五湖三园一池”，新开挖晋安湖、旗山湖，新增调蓄库容520万立方米，“高水高排”工程进展顺利，城区防洪排涝能力明显增强；埋设永久截污管道227公里，修复改造排水管网1116公里，新建雨污水管网146公里，清除内河淤泥256万立方米，打通断头河13条，成立城区水系巡查队伍，城区黑臭水体基本消除；建成生态驳岸70公里、滨河绿道300公里、串珠公园168个，西湖左海实现水通、路通、景通，水系周边环境得到改善；完成水系联排联调信息化平台建设，水系智慧管理体系初步形成；实施连片旧屋区改造54个1133万平方米、老旧小区整治300个、立面景观精准改造2004个；启动建设15个特色历史文化街区，上下杭、朱紫坊历史风貌区部分修复并开街；地铁2号线基本建成即将试运行，地铁4号线、5号线、6号线、滨海快线和1号线二期顺利推进；持续实施392个城区治堵项目；规范早夜市、共享单车和市政设施管理；新建改造公厕924座，建成餐厨垃圾、危险废弃物综合处置场等8个项目；新建公共停车泊位1.2万个、充电桩1500个；全市造林绿化10.9万亩，中心城区种植乔木25万株。新版城市总体规划编制取得进展，“城市双修”总体规划通过评审，闽江两岸规划管控和整治提升工作有效开展。

　　（五）改革开放不断深化。机构改革全面启动，因地制宜设置40个市级政府工作部门，新老机构顺利交替。国有企业改革取得进展，组建电子信息集团、招商服务公司，安泰楼员工持股试点和农垦改革工作稳步推进。流转耕地61万亩，农村土地承包经营权确权颁证进度位居全省前列。福州新区完成重点项目投资2050亿元，三江口、闽江口、福清湾、江阴湾等重点区域和组团加快开发，13项改革任务有效落实。自贸区福州片区推出创新举措27项，8项创新举措在全国复制推广。积极融入“一带一路”战略，“海上福州”建设全面提速，成功获批国家海洋经济发展示范区，罗源湾港区扩大开放通过国家验收，福州港集装箱吞吐量320万标箱，货物吞吐量1.45亿吨；机场一期完成第二轮扩能，新增直飞莫斯科、巴黎等国际航线，空港出入境旅客增长23.5%；成功举办海丝博览会暨第二十届海交会、第四届海丝国际旅游节、第十二届世界福州十邑同乡恳亲大会等重大活动。制定出台68条惠及台胞的具体措施，举办第六届海青节、海峡两岸民俗文化节等43项特色活动。闽东北协同发展区建设扎实推进，福厦客专等50个首批重大合作项目加快建设。榕港澳、闽浙赣皖、泛珠三角区域协作不断拓展。与比利时列日市、毛里塔尼亚努瓦迪布市缔结友城关系。

　　（六）三大攻坚战有效突破。深入防范化解金融风险，设立总规模100亿元的上市企业纾困基金，非法集资、互联网金融等涉众型风险得到稳控，不良贷款实现“双降”。持续巩固脱贫成果，永泰实现省级扶贫县摘帽退出，闽清达到市级扶贫县摘帽退出要求，惠民资金网荣获首届“中国廉洁创新奖”，实施薄弱村发展项目82个，全面消除空壳村和村级组织活动场所“空白点”，援宁援藏援疆、与定西市扶贫协作等工作深入开展。污染防治成效明显，中央、省环保督察反馈问题有效整改，国家生态文明试验区15项改革任务全部完成，商品林赎买、碳汇交易、林地占补平衡等试点工作持续深化，自然资源资产负债表编制及生态产品市场化探索取得突破，河湖长制全面落实，海漂垃圾污染得到较好控制，城市空气质量在全国169个重点城市中排名第8、位居省会城市第3。

　　（七）营商环境得到优化。深化“放管服”改革，全面推行行政执法三项制度和“证照分离”，主导制定国内首个政府部门“双随机一公开”监管工作省级地方标准，完成国务院“互联网+政务服务”综合试点，市级审批服务事项“最多跑一趟”“一趟不用跑”占比分别达93%、31.8%，企业开办、常规不动产登记时间压缩至5个工作日，市政、房建类工程建设项目审批时间压缩至90个工作日以内，进口和出口整体通关时间分别压缩74.2%、85.8%，获得电力平均时长缩短38.6%，制度性交易成本进一步降低，全年减税降费183亿元。推行“一企一议”协调服务机制，帮助企业解决经营发展问题5300多个。成功举办中国城市信用建设高峰论坛，城市信用排名升至全国第7位。

　　（八）民生短板逐步补强。加大民生投入，民生支出723.8亿元，占一般公共预算支出的78.4%。完成20项52件为民办实事项目。新改扩建公办幼儿园13所、中小学41所，普惠性幼儿园学额覆盖率提高至77.3%，与天津大学、新加坡国立大学签订合作办学协议。国家公共文化服务体系示范区创建通过终期验收，全面展开《福州通史》编纂工作，组织文化惠民活动700多场。市妇幼保健院新院、市一医院外科病房大楼等项目加快建设，“榕医通”平台建成投用。在全国率先推出统一电子社保卡，退休人员养老金待遇、城乡居民基础养老金标准、居民医保人均筹资水平稳步提高，城乡低保补助标准实现一体化。新建社区居家养老服务照料中心21个、农村幸福院298所。城镇新增就业13.2万人，转移农业富余劳动力3.8万人。在全国率先推行安置型商品房，动建安置型商品房4万套，建成保障性安居工程1.2万套，解决逾期安置1.1万户。群众体育和竞技体育协调发展，新建全民健身工程项目69个，成功举办环福州·永泰国际公路自行车赛等大型体育赛事，在省运会上获金牌总数、奖牌总数、团体总分“三个第一”。安全生产态势平稳，实行领导干部安全生产责任清单化管理，整治市县两级重大安全隐患点817个，食品药品安全形势稳定向好。社会治安保持稳定，扫黑除恶专项斗争深入推进，平安建设综合评定成绩位居全省前列。社区治理持续完善，军门社区工作法在全国推广，圆满完成村级组织换届选举。全国文明城市、国家卫生城市创建工作取得新成效。民族宗教、信访、行政复议、司法、仲裁以及国防动员、双拥共建、民兵预备役、海防、人防、反走私等工作继续加强。广播影视、新闻出版、哲学社会科学、文学艺术、统计、科普、气象、防震、地方志、档案、老龄、妇女儿童、残疾人、慈善等各项事业全面发展。

　　（九）自身建设切实加强。持续兴起习近平新时代中国特色社会主义思想大学习热潮，“四个意识”更加牢固，“四个自信”更加坚定，“两个维护”更加坚决。认真践行“马上就办、真抓实干”优良作风，深入开展“提振精气神、建设新福州、当好排头兵”主题实践活动。扎实抓好中央巡视反馈问题整改落实。严格贯彻中央八项规定精神，驰而不息整治“四风”。全面落实一线考核、一线巡察机制，持续整治“虚、僵、躲、拖、腐”不良作风，实施效能问责161人次。审计监督力度不断加大。深入推进法治政府建设，依法行政能力持续提升。认真执行市人大及其常委会决定决议，自觉接受人大的法律监督、工作监督。自觉接受市政协的民主监督，支持市政协开展民主协商。提请市人大常委会审议地方性法规草案1件，制定规范性文件23件。办复486件省、市人大代表建议和560件省、市政协提案，满意率分别达99.6%、100%。

　　各位代表，成绩来之不易，这是省委、省政府和市委正确领导的结果，是全市人民团结奋斗、社会各界大力支持的结果。在此，我代表市人民政府，向全市人民，向人大代表、政协委员、各民主党派、工商联、无党派人士、各人民团体、离退休老同志和社会各界人士，向中直、省直机关企事业单位和驻榕部队指战员、武警官兵、公安干警、消防救援队伍指战员，向关心支持福州发展的港澳台同胞、海外侨胞和国际友人，表示衷心的感谢！

　　在总结成绩的同时，我们也看到当前发展中存在的困难和问题，主要是：大项目、好项目不多，产业竞争力不强，创新能力不足，推动高质量发展落实赶超任务还比较艰巨；老城功能品质仍需提升，新城建设步伐尚需加快；改革开放亟待深化，营商环境还需改善，民营经济发展活力有待增强；三大攻坚战任务依然繁重；教育、医疗、住房、养老等民生保障与人民的需要还有差距，等等。对此，我们将采取有效措施，认真加以解决。

　　二、2019年工作安排

　　今年是新中国成立70周年，是全面建成小康社会关键之年。做好今年工作，对推进高质量发展落实赶超、打造幸福之城具有重要意义。根据市委统一部署，今年市政府工作总体要求是：以习近平新时代中国特色社会主义思想为指导，全面贯彻党的十九大和十九届二中、三中全会精神，坚持稳中求进工作总基调，坚持新发展理念，坚持推进高质量发展，坚持以供给侧结构性改革为主线，坚持深化市场化改革、扩大高水平开放，加快建设现代化经济体系，继续打好三大攻坚战，进一步稳就业、稳金融、稳外贸、稳外资、稳投资、稳预期，马上就办、真抓实干，努力建设创新开放绿色幸福的现代化城市，争当新时代新征程上的排头兵。

　　当前，国际形势错综复杂，经济运行稳中有变、变中有忧。但是，从我市情况看，“五区叠加”的战略机遇没有变，经济发展稳中向好的态势没有变。我们既要增强忧患意识，又要保持战略定力，更要树立必胜信心，一心一意谋发展，集中精力抓建设。今年发展的主要预期目标是：地区生产总值增长8.6%，地方一般公共预算收入增长4%，固定资产投资增长11%，社会消费品零售总额增长11%，进出口总额和实际利用外资增速高于省里目标，居民消费价格总水平涨幅控制在3%以内，城镇居民人均可支配收入增长7.5%，农村居民人均可支配收入增长8.5%，城镇登记失业率控制在3.5%以内，完成节能减排降碳任务。

　　为实现上述预期目标，重点抓好以下四个方面工作：

　　（一）打造创新福州，全面推动经济高质量发展。

　　始终把创新摆在发展的核心位置，开展“项目年”专项行动，抓项目、补链条、强产业，加速新旧动能转换，加快构建现代化经济体系。

　　培育壮大新兴产业。加快中科光芯、京东方柔性显示等项目建设，推动新一代信息技术产业增“芯”强“屏”；完善健康医疗大数据、基因检测、分子诊断产业布局，做大生物医药产业；加快博那德一体化、吉特瑞医用再生材料等项目建设，做大新材料产业；扶持启瀚新能源、雪人股份、金风科技等项目建设，做大新能源产业；推动中铝瑞闽汽车轻量化、东南汽车新能源乘用车等项目落地投产，做大新能源汽车产业。鼓励企业加大研发投入，支持“知创福建”公共服务平台建设，新增知识产权示范优势企业50家。实施高新技术企业倍增工程，新增省级以上高新技术企业200家、科技小巨人领军企业50家。推进国家自主创新示范区福州片区建设，发挥国家双创示范基地、海西创业基地、中科院海西研究院等载体作用，激发高新区创新活力。

　　实施数字经济领跑。加快“数字福州”建设，推动云计算、大数据、物联网、人工智能、区块链等新一代信息技术和实体经济深度融合，力争数字经济占GDP比重达37.5%以上。高水平办好第二届数字中国建设峰会，积极创建国家数字经济示范区；加强国家级互联网骨干直联点监测管理，发挥数字福建“两朵云”辐射带动作用；做大做强东南大数据产业园，推进字节跳动东南营销中心、皮皮虾产品线和京东元洪食品数字经济产业中心等项目建设；提升马尾物联网产业基地，推广应用智能水务商用项目，在窄带物联网共性技术、通信服务等方面实现突破；建设旗山湖“智谷”、永泰人工智能小镇，建成福建省人工智能超算平台二期；筹建比特大陆区块链研究中心，打造区块链经济综合试验区；加快福州软件园扩容和分园建设，推进数字经济产业集群发展。

　　促进传统产业升级。持续实施扶持技改策略，落实智能、集成、融合技改项目100个，完成技改投资750亿元、增长15%。持续实施扶持上市策略，发挥上市引导基金作用，推动10家以上企业进入上市程序。持续实施品牌强市策略，打造一批具有国际影响力和竞争力的名企、名牌。持续实施建链、强链、补链工程，加快缘泰化工新材料、中景聚丙烯等项目建设，支持龙头企业并购优质企业、营销渠道、关键设备，延伸补齐产业链。加大低效工业用地处置力度，力促腾笼换鸟、产业集聚，提升工业园区发展水平。推进市属国有企业产业化、市场化进程，设立国有企业产业发展基金，组建市商贸集团和建工集团。开展人才聚榕提质行动，办好“榕博汇”，促进高校毕业生在榕就业创业。

　　推动第三产业跃升。开展“三产年”专项行动，培育大宗商品交易平台、营利性服务业企业、批发销售中心、工业企业供应链等新增长点，力争三产增加值增长10.2%。培育网络批发零售、行业性定价结算等公共平台，促进大平台、大市场、大流通融合发展。做强闽江北岸中央商务区等高端总部集聚区，新培育企业总部50家以上。做活东街口、苏万宝、浦上等一、二线商圈，培育消费新业态、新模式，建设中国新零售之都。推进全国首批供应链体系重点城市建设，打造区域性物流集散中心，创建国家级网络市场监管与服务示范区。加快发展服务型制造，新增制造业主辅分离企业20家以上。发展科技金融、供应链金融、绿色金融，壮大海西现代金融中心区。发挥上市企业纾困基金作用，防控涉众型金融风险，打好防范金融风险攻坚战。完成鼓岭国家级旅游度假区改造提升，推进“旅游+”产业融合发展，创建国家全域旅游示范区。办好中国高等教育博览会等大型展会，大力发展会展经济。

　　发展特色现代农业。落实粮食安全省长责任制，确保粮食种植面积稳定在150万亩以上。发展设施农业，实施现代种业工程，新增设施农业2500亩以上。发展休闲农业，建成市级休闲农业公共信息服务平台，培育5条以上休闲农业精品线路。发展品牌农业，新增22个“三品一标”农产品，培育10个“一村一品”示范村。发展农产品精深加工，推进元洪国际食品产业园、鉴江现代渔业产业园等项目建设。发展农业产业园区，培育30个农产品标准化生产示范基地。发展远洋渔业，拓展境外养殖、深海养殖，申报国家远洋渔业基地，持续办好渔业周·渔博会。

　　（二）打造开放福州，持续增强发展活力动力。

　　贯彻落实习近平总书记在庆祝改革开放40周年大会上的重要讲话精神，主动融入国家开放大局和“一带一路”战略，汇集更多资源，推动我市新一轮发展。

　　构建全面开放新格局。开展“招商年”专项行动，持续实施“回归工程”，强化产业链招商、技改招商、服务业招商，试点产业基金招商，推动福南宁区域协同招商合作。发挥福州新区先行先试功能，在三区融合、金融创新、区域综合开发、重大平台建设等方面积极探索，提高开放开发水平。办好海丝博览会暨第二十一届海交会、第六届丝路国际电影节、第五届海丝国际旅游节等活动，抓好中日“一带一路”合作国际示范区筹备工作，推进“海上丝绸之路：福州史迹”申报世界文化遗产，深化海丝战略支点城市建设。落实68条惠及台胞的具体措施，办好第七届海青节，加强台湾青年就业创业基地建设，加快复建台湾会馆，推动马尾琅岐对台客运码头投入运营，促进榕马率先融合发展，为夯实和平统一基础多作示范、多作贡献。组织侨情普查，加强华人华侨新生代工作，强化与港澳在金融、物流、文化创意及青少年等领域的交流。发挥省会城市在闽东北协同发展中的带动服务作用，深化泛珠三角、闽浙赣皖区域协作，继续抓好与渭南、延安、吉安对口合作。

　　建设自贸试验区改革开放新高地。注重“首创性”探索，先行先试50项改革创新措施。鼓励企业到“一带一路”沿线设立海外仓，大力发展跨境电商、离岸贸易等新型业态，力争获批国家跨境电商综合试验区。引进大型龙头车企，做大青口汽车城和银河国际汽车园，打造东南区域进口汽车集散中心。持续发布金融创新案例，推动海峡基金业综合服务平台落地，探索区块链金融服务创新。落实新版负面清单，争取国家惠台政策在自贸区率先落地实施，建立双向投资综合服务体系。推进中国·印尼“两国双园”建设。力争开通中欧（福州）班列，积极参与“丝路海运”。

　　深化“海上福州”建设。推进江阴港6—9号泊位、可门港6—7号泊位、松下港12—13号泊位建设，完善提升江阴港集装箱码头、罗源湾散货码头，加快福州港口后方铁路通道前期工作，鼓励航运企业增开国际航线，建设大宗商品、整车进口等仓储集散中心，推动福州港集装箱吞吐量快速增长，打造国际深水大港。加快江阴化工新材料专区、罗源湾南岸临港产业基地、罗源湾北岸冶金产业基地、松下港粮油肉类加工产业基地建设，做大蓝色经济产业园等特色园区，发展临港产业。优化海洋资源要素市场化配置，打造“海洋牧场”，建设“智慧海洋”，推进国家海洋经济发展示范区建设。全面治理海漂垃圾，推广生态岛礁工程，合理开发海岛资源，保护修复海洋生态。

　　打造一流营商环境。全面完成市县两级机构改革，构建优化协同高效的机构职能体系。持续深化“放管服”改革，加强政务信息整合共享，市县两级政务服务事项网上可办率达90%以上，“一趟不用跑”事项达35%以上，企业开办时间压缩到3个工作日以内，水利、交通类工程建设项目审批压缩到90个工作日以内，降低诉讼“执行合同”的时间和成本，完善国际贸易“单一窗口”模式，进一步压缩通关时间，降低口岸收费。加大“首台套”等惠企政策落实力度，完善“一企一议”协调服务机制，常态化为企业排忧解难。健全社会信用体系，扩大“信用+”运用范围，强化失信行为惩戒。落实国家统计制度改革，做好第四次全国经济普查。

　　扶持民营经济发展壮大。坚持“两个毫不动摇”“三个没有变”，落实好36条民营企业扶持措施，实施市场准入负面清单制度，营造公平竞争环境。落实减税降费政策，清理、精简涉及民间投资的行政审批事项和涉企收费，实质性减轻企业负担。加大融资增信支持，发挥政策性融资担保机构作用，鼓励民营企业通过资本市场直接融资。畅通民营企业困难诉求渠道，维护企业合法权益。推进混合所有制经济发展，鼓励民营企业参与国有企业重组改制。

　　（三）打造绿色福州，切实优化城乡宜居环境。

　　践行绿色发展理念，坚持新城建设、老城提升、乡村振兴同步推进，提升品质内涵，促进城乡一体化发展。

　　加强城市管理。坚持规划引领，加快全市国土空间规划编制和重点区域城市设计。实施宜居社区、园林绿化、公共设施配套等130个示范项目，完成“城市双修”试点工作。健全完善网格化管理系统和市容市貌精细化管理机制，加快市政设施管养、道路清扫保洁市场化进程。推进生活垃圾分类投放、收集、运输和处置工作，力争生活垃圾回收利用率达30%以上。建成大件垃圾处置厂、垃圾焚烧发电三期等4个项目，建设生活垃圾焚烧协同处置、厨余垃圾处理厂，打造红庙岭循环经济产业园。继续推行“厕所革命”，新改建城乡公厕318座。持续治理“两违”、非法采砂等问题。

　　改善城市面貌。增强城市承载能力，加快地铁4号线、5号线、6号线、滨海快线和1号线二期建设，地铁2号线5月1日投入试运营；建成绕城高速东南段、长平高速、马尾大桥、南台大道等64个项目，提速福厦客专、莆炎高速、城区北向第二通道、洪塘大桥等58个项目，新改扩建市政道路124公里；新建改造供水管网180公里、燃气管网150公里，建成城区双气源、双通道供气系统。开展城市道路畅通行动，继续实施城区258个治堵项目，新增公共停车泊位7500个、新能源公交车200辆，改造公交停靠站100个。开展水环境综合整治扫尾行动，城区水系全面转入常态化运维管养；加快“高水高排”工程建设，建成晋安河直排闽江通道；新建改造雨污水管网1000公里；再建生态驳岸17.5公里、滨河绿道100公里、串珠公园50个；建成城区水系科学调度系统，实现内河水系智慧管理；完成鹤林、三江口片区海绵城市建设试点任务。开展老城提升行动，改造连片旧屋区52个1206万平方米，整治老旧小区60个，持续推进缆线下地。继续开展“全民动员、绿化福州”，创建国家生态园林城市。大力推广绿色建筑、装配式建筑。建立棚改和水系治理资金智慧化管理平台。

　　加快乡村振兴。因地制宜，分类推进乡村振兴初级版、中级版、高级版规划建设，培育乡村振兴试点镇30个、试点村300个。实施“一革命、五行动”，开展“村植千树”活动，整治农房2000栋以上，提升村庄生活垃圾无害化处理水平，实现行政村污水治理、卫生厕所全覆盖，打造美丽乡村300个、美丽乡村示范村100个；推进“四好农村路”建设，继续抓好高速公路、高铁沿线人居环境整治。传承保护乡村文化，加强历史文化名镇、传统村落、民族特色村寨等保护修复。深化农村改革，完成土地承包经营权确权颁证，引导农村土地有序流转，打造10个500亩以上土地流转相对集中片区；深化农村集体产权、集体林权、国有林场、农垦等改革。设立村级乡村振兴服务站2191个，选派省市科技特派员600名以上。支持闽清、永泰创建国家级农村电商示范县。推动福清国家级新型城镇化综合试点建设。

　　提速新城建设。全面加快长乐滨海新城建设，编制松下分区等7项规划，实现新城核心区控制性详规全覆盖；加快东南快速通道等基础设施建设，基本建成启动区骨干路网；建成数字中国会展中心、福州三中滨海校区等60个项目，动建中央商务区、东湖湿地公园等50个项目，完成年度投资450亿元以上；加快机场第二跑道前期工作，力争年底动建；建设国家级邮轮旅游发展实验区；争取获批福州空港综合保税区。高标准建设三江口片区，加快樟岚总部集聚区、道庆洲过江通道、马航洲湿地保护修复工程等69个项目建设。加快琅岐岛综合开发，完善岛内基础设施，建成投用酒店、乐园、农庄、博物馆等首批项目。通过设立分院分校、市建省管、市县共建等方式，推动教育、医疗、文化等优质资源向新城辐射。

　　强化生态保护。推进生态文明试验区建设，推出一批可复制可推广的创新举措。探索自然资源资本化路径，做好连江生态产品市场化试点。实施生态环境损害赔偿制度，推进大樟溪流域综合性生态保护补偿试点。开展党政领导干部自然资源资产离任审计，健全自然资源资产负债表。强化挥发性有机物排放、机动车尾气、施工工地和道路扬尘管控，确保空气质量保持全国前列。实施闽江流域（福州段）山水林田湖草生态保护修复工程，打好敖江、龙江流域综合治理攻坚战。持续开展水土流失治理，完成市县两级集中式饮用水水源地环境整治。继续实施危险废物污染防治工程包，开展土壤污染风险防控试点。做好第二次全国污染源普查、第三次全国国土调查工作。

　　（四）打造幸福福州，有效保障和改善民生。

　　坚持以人民为中心的价值取向，多办利民实事，多解民生难事，切实增强人民群众的获得感、幸福感、安全感。

　　提高教育供给质量。努力破解普惠性学前教育供给不足、“择校热”、“大班额”等问题，新改扩建幼儿园20所，新增示范幼儿园42所，普惠性幼儿园学额覆盖率达80%以上；新改扩建中小学37所，推动15所小学、5所初中实施学校联盟、集团化办学。加强普高课改基地校、达标校建设，全面实施高考综合改革。引导职业院校增设产业发展急需专业，培育省级示范性中职学校7所。推动市属本科院校建设高水平应用型大学，加快闽江学院更名进程。推进与天津大学、新加坡国立大学、清华附中、北大附中等名校合作。探索解决中小学教师编制不足问题，推进中小学教师“县管校聘”改革。加强师德师风建设和专业培训，改进中小学课后服务。提升继续教育、老年教育、社区教育和特殊教育办学水平，加快建设学习型城市。

　　促进文化繁荣发展。加强闽都文化传承和弘扬，加快新店古城遗址公园和冶山、于山、鼓岭等历史风貌区保护利用，提速南公园、梁厝村等15个特色历史文化街区建设，启动73条传统老街巷保护整治。培育“文化+旅游”“文化+科技”等文创新业态，加强“文化惠民·六进”等公共文化品牌建设，加快群众艺术馆新馆、科技馆新馆、少儿图书馆新馆、商务印书馆福州分馆规划建设，动建非遗展示馆，提升美术馆新馆。办好第十六届中国戏剧节和首届闽剧节。振兴传统工艺美术。促进哲学社会科学、新闻出版、广播影视、文学艺术等事业繁荣发展。

　　加强健康福州建设。支持省儿童医院、妇产医院等省级项目加快建设进度，启动市中医院分院、市皮肤病院医技楼、市一医院门诊医技楼等5个项目。实施社区卫生服务中心能力提升工程，建设100个标准化村卫生所。深化医保支付和药品采购方式改革，加快紧密型医联体建设。完善公共卫生应急体系。持续开展爱国卫生运动。建成2个全国名老中医药专家传承工作室。完善全面两孩政策配套措施，做好儿童青少年近视防控工作，强化妇幼健康服务保障。改造提升56个全民健身工程、250个智能化健身路径，办好举重世界杯赛、中华龙舟大赛等大型赛事，打造一批群众性赛事品牌。

　　增强社会保障能力。稳步扩大社会保险覆盖面，提高城乡居民医保财政补助标准。实现社区居家养老服务照料中心、市县两级社会福利中心全覆盖，实现农村幸福院覆盖率达65%以上。大力发展社会救助、社会慈善和残疾人事业。创新殡葬管理模式，推行惠民殡葬。加快构建租购并举的住房制度，完善多层次住房保障体系，基本解决历史遗留的逾期回迁安置问题。抓好“菜篮子”工程，全方位创建国家食品安全示范城市，用“四个最严”捍卫市民舌尖上的安全。拓宽居民劳动收入和财产性收入渠道，增加低收入群体收入。做好重点群体就业帮扶，城镇新增就业12.2万人，转移农业富余劳动力3万人，城镇失业人员再就业8500人。健全完善工资支付诚信等级评价制度，构建和谐劳动关系。

　　巩固脱贫攻坚成果。完善稳定脱贫长效机制，落实健康扶贫、教育扶贫、就业扶贫和低保兜底政策，强化驻村帮扶、产业扶贫和资产收益扶贫等措施，确保脱贫户持续增收。大力发展村级集体经济，扶持80个薄弱村发展现代农业。支持革命老区、少数民族村发展特色经济，增强“造血功能”。灵活用好扶贫发展基金，充分调动社会各方力量参与扶贫事业。发挥惠民资金网作用，提高扶贫资金使用效益。继续抓好援宁援藏援疆工作，深化与定西市的扶贫协作。

　　推动社会治理创新。深入实施城乡社区治理三年行动，推进“雪亮工程”、网格化管理平台、综治中心一体化建设，扎实推动扫黑除恶专项斗争向全面纵深发展，推进“一县（市）区一特色品牌”普法示范点建设，实现县级社区矫正中心全覆盖，完善社会治安防控体系，打造高水平的“平安福州”。健全防灾减灾救灾机制，完善安全生产责任体系，守住不发生重特大安全事故的底线。巩固双拥模范城创建成果，做好退役军人安置、管理和保障工作，推进军民融合深度发展。加强国防教育、国防动员、海防、人防、反走私和民族宗教等工作。支持工会、共青团、妇联、残联等人民团体发挥更大作用。做好应急、统计、科普、保密等工作。

　　建设现代文明城市。大力践行社会主义核心价值观，持续深化“有福之州·文明同行”活动，深入开展文明城市、文明村镇、文明单位、文明校园、文明家庭创建工作，加强“讲文明树新风”公益广告宣传，提升市民文明素质。开展中小学“扣好人生第一粒扣子”主题教育实践活动，抓好青少年行为习惯养成教育，促进未成年人健康成长。建设志愿服务孵化基地，深化志愿者服务行动。推进新时代文明实践中心试点建设，巩固提升移风易俗成果，倡导文明乡风、良好家风、淳朴民风。

　　三、建设对党忠诚、人民满意的服务型政府

　　各位代表，新时代新征程新任务对政府自身建设提出了新的更高的要求。我们将进一步加强学习，提高站位，锐意创新，担当作为，不断开创政府工作新局面。

　　加强政治建设。持续深入学习习近平新时代中国特色社会主义思想和党的十九大精神，牢固树立“四个意识”，坚定“四个自信”，坚决维护习近平总书记在党中央的核心、全党的核心地位，坚决维护党中央权威和集中统一领导。坚决服从市委在推动福州发展中总揽全局、协调各方的领导作用，以实际行动确保党中央国务院、省委省政府和市委各项决策部署落到实处。

　　坚持依法行政。严格遵守宪法法律，把政府活动全面纳入法治轨道。坚持严格规范公正文明执法，有权不可任性，用权必受监督。依法接受市人大及其常委会的监督，自觉接受人民政协的民主监督，主动接受社会和舆论监督，认真听取各民主党派、工商联、无党派人士和各人民团体、社会各界人士的意见建议，提高办理人大代表建议、政协提案和协商意见的落实率、满意率。

　　强化担当作为。坚决贯彻市委进一步提振干部干事创业精气神的要求，落实一线考核、正向激励、容错纠错机制，为负责任的干部负责，为敢担当的干部担当。坚持“马上就办、真抓实干”，压紧夯实责任，层层传导压力，激发干部干事创业新动力，营造比学赶超、竞相发展的良好氛围。

　　提升服务水平。把广大企业、群众反映强烈的突出问题作为工作重点和突破口，坚决破除一切阻碍，大力推行一线工作法、项目工作法，用脚步丈量民情，靠两条腿走出项目，把办公地点落到一线，把协调会场放到一线，在一线推动工作，在一线解决问题。创新体制机制，改进工作方法，构建更加便捷高效的服务体系。

　　打造廉洁政府。坚决扛起全面从严治党主体责任，全面加强党风廉政建设，把权力关进制度的笼子，对各类腐败行为坚持零容忍。严格贯彻中央八项规定精神，持续推进扶贫领域、民生领域、涉黑涉恶腐败、移风易俗等四个专项治理，重拳整治群众身边的腐败和作风问题。强化审计监督，落实审计整改责任，严明财经纪律。建立政企直通车等服务制度，构建新型亲清政商关系。

　　各位代表，新征程既有机遇又有挑战，新目标需要全市上下一起拼搏、一起奋斗。让我们更加紧密地团结在以习近平同志为核心的党中央周围，高举习近平新时代中国特色社会主义思想伟大旗帜，在省委、省政府和市委的坚强领导下，坚定信心、鼓足干劲，携手并肩、勇往直前，建设有福之州、打造幸福之城，以优异成绩庆祝新中国成立70周年！
